# Dental caries and related factors in the elderly of the Azar cohort population: A cross-sectional study

**DOI:** 10.1371/journal.pone.0315725

**Published:** 2025-01-15

**Authors:** Maryam Hosseinpour Sarmadi, Nasrin Sharififard, Zeinab Mahboobi, Elnaz Faramarzi, Aylin Bilehjani

**Affiliations:** 1 Liver and Gastrointestinal Diseases Research Center, Tabriz University of Medical Sciences, Tabriz, Iran; 2 Faculty of Dentistry, Department of Oral and Maxillofacial Medicine, Tabriz University of Medical Sciences, Tabriz, Iran; 3 Faculty of Dentistry, Department of Community Oral Health, Tabriz University of Medical Sciences, Tabriz, Iran; 4 Faculty of Dentistry, Tabriz University of Medical Sciences, Tabriz, Iran; Shahid Beheshti University of Medical Sciences School of Dentistry, ISLAMIC REPUBLIC OF IRAN

## Abstract

**Objective:**

Oral health is often overlooked among the elderly due to the numerous comorbidities prevalent in this population. However, oral health significantly influences quality of life by affecting both general health and psychological well-being. The present study aimed to assess dental caries in elderly individuals using the DMFT index (decayed, missing, and filled teeth) and to explore its relationship with various factors based on data from the Azar cohort study in Iran.

**Materials and methods:**

In this cross-sectional study, data from the initial phase of the Azar cohort study, which involved 2629 elderly individuals (aged 60 years and older), were statistically analyzed. The Azar cohort study evaluated demographic factors, the history of chronic diseases, and behavioral habits using a well-designed questionnaire administered through face- to -face interviews. The DMFT index was evaluated through oral examination. A negative binomial regression analysis with a log link function was employed to investigate the relationship between the DMFT and related variables, including gender, age, marital status, level of education, socioeconomic status, chronic diseases, smoking, alcohol consumption, and body mass index.

**Results:**

The mean (SD) age of elderly individuals was 64.15±2.91 years. The mean (SD) DMFT was 28.42±6, and the mean (SD) number of missing teeth was 26.58±8.36. Approximately 70.8% of elderly individuals were edentulous. Women exhibited a higher mean DMFT score and a greater number of missing teeth compared to men. The mean DMFT score and its components significantly differed based on the level of education and socioeconomic status. However, no significant relationship was found between the DMFT index and the variables in the multiple regression analysis.

**Conclusion:**

The high prevalence of edentulism and the elevated DMFT scores in the elderly population of the Azar cohort indicate a poor oral health status among older individuals. Providing the dental services within the primary health care system for the adults, can be beneficial in improving oral health in old age.

## Introduction

There are approximately one billion elderly individuals aged 60 and olderworldwide, a number projected to 1.4 billion by 2030 and 2.1 billion by 2050 [1]. Aslife expectancy increases and people live longer, the prevalence of oral and dental issues is likely to rese [2]. Oral health is crucial for the quality of life among elderly individuals [3]. It influences social interactions, dietary choices, body weight, as well as the ability to eat and speak [4]. In some cities in Iran and other countries, the oral health of elderly individuals is concerning, make it a significant public health issue that requires greater global attention [5–9].

The factors influencing the oral health status of elderly individuals vary across different communities due to cultural, social, and economic disparities [10]. Studies conducted in Brazil [8], Germany [9], and Italy [11] reported the DMFT scores for elderly individuals as 28.16, 26.4, and 13.8, respectively. A national dental survey conducted in Iran in 2013 indicated that the mean DMFT for elderly individuals was 25.71 [12]. Additionally, studies in Isfahan [13], Tehran [14], and Yazd [7] reported DMFT score for elderly individuals as 20.5, 22, and 26.6, respectively.

While some studies in Iran have explored the relationship between oral health status, quality of life, and demographic factors among elderly individuals [5,6], there is a lack of comprehensive research on the association of various factors with the DMFT index in this population. This study aims to evaluatedental cariesamong the elderly in Iran using the DMFT index and to examine its correlation with various factors based on data from the Azar cohort study.

## Materials and methods

This research is a cross-sectional study based on baseline data collected from the Azar cohort study, which focuses on elderly aged 60 to 70 years.

In the Azar cohort study conducted in 2014, 15,006 adults aged 35 to 70 years were selected from the permanent residents of Shabestar city in East Azerbaijan Province, northwest Iran. The Azar Cohort was part of the Prospective Epidemiological Research Studies in Iran (PERSIAN cohort), which aimed to assess the risk factors for noncommunicable diseases [15–17]. Evaluators conducted interviews using predefined and validated research protocols, along with Persian cohort questionnaires [16]. The objectives of the Azar cohort, the sampling method, the variables, and the data collection tools and methods have been detailed in previously published articles [16,17]. At the time of enrollment, written informed consent was obtained from participants, or their legal guardian in the case of illiterate participants. Those who completed the informed consent process were included in the study, with the option to withdraw at any time and for any reason. To ensure anonymity, participants in the Azar cohort were assigned unique identification codes. In this cross-sectional study, data from all elderly individuals aged 60 to 70 years whitin the Azar cohort population (n = 2,629) were included without any exclusions.

In the present stud, we analyzed data concerning, DMFT index, gender, age, marital status, level of education, socioeconomic status, history of chronic diseases, smoking habits, alcohol consumption, frequency of tooth brushing, and body mass index.

The DMFT index which represents the total number of decayed, missing, and filled teeth, serves as the outcome variable for this study. It is classified as a quantitative discrete variable. A dental examination was conducted by a single skilled examiner using a headlight, intraoral mirror, and explorer in accordance with the World Health Organization (WHO) Oral Health Surveys Basic Methods [18,19] to record the DMFT index. The components of the DMFT index -D (decayed teeth), M (missing teeth), and F (filled teeth)- were analyzed separately.

The data from the Azar cohort Questionnaires were extracted as follows: The frequency of tooth brushing was categorized into two groups: brushing at least once a day and brushing less frequently than once a day.

Age, as a qualitative variable, was categorized into two groups: 60–64 years and 65–70 years. Gender was also examined as a qualitative variable, divided into two groups: men and women. Marital status was classified into two categories: unmarried and married. Socioeconomic status was assessed using the Wealth Score Index (WSI), which was calculated through Multiple Correspondence Analysis (MCA). The WSI for each participant was determined based on ownership of various durable assets (e.g., dishwasher, car, and television), household conditions (e.g., number of rooms, type of ownership), and educational attainment. Participantsof the study were classified into five WSI quintiles, ranging from the lowest (1st quintile) to the highest (5th quintile): very poor, poor, average, good, and very good. The level of education was categorized into four groups: illiterate, primary, diploma and university. The smoking was grouped into three categories: non-smoker, ex-smoker, and smoker. The Body mass index (BMI) was analyzed as a qualitative variable with four categories: <18.5, 18.5–24.9, 25–29.9, and ≥30 kg/m^2^. Alcohol consumption was classified as qualitative variable with two responses: Yes and No. Additionally, self-reported histories of chronic diseases were extracted from the Azar cohort data. This cross-sectional study received approval from the Ethics Committee of Tabriz University of Medical Sciences, Tabriz, Iran (IR.TBZMED.REC.1401.527).

### Statistical analysis

Statistical analysis was conducted using SPSS version 16 software (IBM Company, Chicago, IL, USA). Asignificance level of p<0.05 was established. Following the normality test, which utilized skewness and kurtosis indices, t-tests and one-way ANOVA were applied to compare the mean DMFT and M values. The Mann‒Whitney U test and Kruskal‒Wallis test were used to compare the D and F values based on the specified variables. Negative binomial regression with a log link function was employed to adjust for the effects of all potential confounding variables.

## Results

In the present study, data collected from 2,629 elderly individuals aged 60 to 70 years in the Azar cohort study were analyzed to examine the relationship between the DMFT index and various factors. The average age of the participants was 64.15±2.91 years. Approximately 52% of the elderly participants were women. A high body mass index was reported in 76% of the elderly population. The frequencies of all variables are presented in Table 1.

**Table 1:** Characteristics of the elderly in the Azar cohort population based on the considered variables.

Variable	Category	N	%
**Age**	60–64	1525	58
	65–70	1104	42
**Gender**	Male	1257	47.8
	Female	1372	52.2
**Marital status**	unmarried	393	14.9
	Married	2236	85.1
**Level of education**	Illiterate	1154	43.9
	Primary	736	28
	Diploma	637	24.2
	University	102	3.9
**WSI** **(Wealth Score Index)**	Very poor	882	33.54
	Poor	676	25.71
	Medium	526	20.01
	Good	252	9.6
	Very good	293	11.1
**Diabetes mellitus**	No	2059	78.3
	Yes	570	21.7
**Hypertension**	No	1467	55.8
	Yes	1162	44.2
**Stroke**	No	2571	97.8
	Yes	58	2.2
**Cardiovascular diseases**	No	2313	88
	Yes	316	12
**Cancer**	No	2598	98.8
	Yes	31	1.2
**Depression**	No	2177	82.8
	Yes	452	17.2
**Chronic obstructive pulmonary diseases**	No	2467	93.8
	Yes	162	6.2
**Smoking**	Non smoker	1987	75.6
	Ex-smoker	333	12.7
	Smoker	309	11.8
**BMI** **(Body mass index) kg/m2**	18.5>	25	1
	18.5–24.9	587	22.3
	25–29.9	1072	40.8
	≥30	945	35.9
**Alcohol consumption**	No	2564	97.5
	Yes	65	2.5

The mean (SD) DMFT score was 28.42±6. The means (SD) for the D, M,and F components were 1.28±3.42, 26.58±8.36, and 0.55±2.13, respectively. Approximately 70.8% of the elderly participants were completely edentulous, and 64.4% of them brushed their dentures at least once a day. Only 28% of the elderly individulas with natural teeth brushed their teeth at least once a day.

The mean DMFT score and the number of missing teeth were significantly higher in individuals aged 65 to 70 years compared to those aged 60 to 64 years (P<0.001). Conversly, the mean number of filled teeth in the 60 to 64 age group was significantly greater than that in the 65 to 70 age group (P<0.001). additionally, women exhibited a higher DMFT and a greater number of missing teeth (P = 0.003, P<0.001) compared to men. However, the mean number of decayed teeth was significantly higher in men (P<0.001).

The mean DMFT score and its components demonstrated a significant difference based on the level of education (P<0.001). The DMFT score was higher in the illiterate group compared to the elementary group (P = 0.01). Furthermore, DMFT was greater in the illiterate group than in both the diploma and university groups (P<0.001). The mean number of missing teeth was higher among individuals with lower education levels, while the mean number of filled teeth was greater among those with higher education levels. Individuals with very poor socioeconomic status exhibited a higher DMFT score than those with good or very good socioeconomic status (P<0.001). The mean DMFT score among smokers was significantly higher (P<0.001). Moreover, the mean DMFT score was significantly greater in elderly individuals with a lower BMI (P = 0.04). However, the DMFT score in the elderly did not show a significant difference based on alcohol consumption. A comparison of DMFT scores based on various factors is presented in Table 2.

**Table 2:** Comparison of the mean DMFT score based on considered variables in elderly subjects of the Azar cohort population (n = 2629).

Variable	Category	DMFT *		D**		M*		F**	
		Mean±SD	P	Mean±SD	P	Mean±SD	P	Mean±SD	P
**Age**	**60–64**	**27.97±6.34**	**<0.001**	**1.29±3.28**	**0.003**	**25.99±8.73**	**<0.001**	**0.69±2.37**	**<0.001**
	**65–70**	**29.04±5.43**		**1.28±3.60**		**27.40±7.75**		**0.36±1.71**	
**Gender**	**Male**	**28.04±6.28**	**0.002**	**1.84±4.15**	**<0.001**	**25.57±8.89**	**<0.001**	**0.62±2.24**	**0.02**
	**Female**	**28.77±5.71**		**0.78±2.47**		**27.50±7.73**		**0.49±2.01**	
**Marital status**	**Unmarried**	**28.69±5.57**	**0.33**	**1.02±2.88**	**0.19**	**27.34±7.59**	**0.05**	**0.33±1.47**	**0.11**
	**Married**	**28.37±6.07**		**1.33±3.50**		**26.45±8.49**		**0.59±2.22**	
**Educational level**	**Illiterate**	**29.28±5.24**	**<0.001**	**1.09±3.17**	**<0.001**	**28.05±7.20**	**<0.001**	**0.14±0.89**	**<0.001**
	**Primary**	**28.57±5.94**		**1.68±4.08**		**26.49±8.39**		**0.40±1.80**	
	**Diploma**	**27.44±6.45**		**1.24±3.15**		**25.10±9.06**		**1.10±3.00**	
	**University**	**23.57±8.09**		**0.87±1.94**		**19.78±10.68**		**2.91±4.34**	
**WSI** **(Wealth Score Index)**	**Very poor**	**28.80±5.70**	**<0.001**	**1.58±3.92**	**0.25**	**26.97±8.10**	**<0.001**	**0.25±1.26**	**<0.001**
	**Poor**	**28.93±5.52**		**1.04±2.86**		**27.51±7.56**		**0.38±1.73**	
	**Medium**	**28.27±6.21**		**1.44±3.77**		**26.18±8.73**		**0.64±2.31**	
	**Good**	**27.89±6.31**		**1.15±3.10**		**25.99±8.55**		**0.75±2.36**	
	**Very good**	**26.78±6.93**		**0.78±2.28**		**24.48±9.59**		**1.52±3.63**	
**Diabetes mellitus**	**No**	**28.51±5.93**	**0.10**	**1.28±3.42**	**0.51**	**26.70±8.29**	**0.16**	**0.53±2.12**	**0.05**
	**Yes**	**28.06±6.22**		**1.28±3.41**		**26.15±8.58**		**0.62±2.12**	
**Hypertension**	**No**	**28.49±5.86**	**0.43**	**1.46±3.70**	**0.005**	**26.52±8.33**	**0.65**	**0.52±2.01**	**0.83**
	**Yes**	**28.31±6.16**		**1.06±2.99**		**26.66±8.45**		**0.59±2.25**	
**Stroke**	**No**	**28.41±6.00**	**0.94**	**1.29±3.43**	**0.86**	**26.57±8.37**	**0.68**	**0.56±2.14**	**0.93**
	**Yes**	**28.36±6.17**		**1.03±2.57**		**27.02±8.04**		**0.31±1.35**	
**Cardiovascular diseases**	**No**	**28.43±5.96**	**0.74**	**1.32±3.47**	**0.05**	**26.55±8.36**	**0.56**	**0.56±2.16**	**0.79**
	**Yes**	**28.31±6.23**		**0.98±2.94**		**26.83±8.34**		**0.50±1.84**	
**Cancer**	**No**	**28.42±5.99**	**0.40**	**1.29±3.43**	**0.95**	**26.60±8.35**	**0.38**	**0.54±2.09**	**0.004**
	**Yes**	**27.51±6.63**		**0.68±1.5**		**25.29±8.77**		**1.55±3.92**	
**Depression**	**No**	**28.39±6.04**	**0.68**	**1.35±3.52**	**0.08**	**26.50±8.41**	**0.32**	**0.54±2.11**	**0.26**
	**Yes**	**28.52±5.80**		**0.98±2.83**		**26.94±8.13**		**0.61±2.16**	
**Chronic obstructive pulmonary diseases**	**No**	**28.39±6.03**	**0.54**	**1.30±3.43**	**0.27**	**26.54±8.40**	**0.34**	**0.56±2.16**	**0.47**
	**Yes**	**28.69±5.53**		**1.12±3.26**		**27.18±7.61**		**0.40±1.54**	
**Smoking**	**Non-smoker**	**28.24±6.20**	**<0.001**	**1.21±3.25**	**0.15**	**26.45±8.54**	**0.002**	**0.57±2.21**	**0.03**
	**Ex-smoker**	**28.04±5.99**		**1.53±4.00**		**25.93±8.38**		**0.58±1.80**	
	**Smoker**	**29.99±4.22**		**1.46±3.75**		**28.10±6.93**		**0.43±1.90**	
**BMI (kg/m** ^**2**^ **)**	**>18.5**	**30.84±2.85**	**0.04**	**1.68±4.43**	**0.52**	**29.16±6.07**	**0.20**	**0.00±0.00**	**0.17**
	**18.5–24.9**	**28.80±5.61**		**1.28±3.43**		**26.99±8.10**		**0.53±2.08**	
	**25–29.9**	**28.39±6.11**		**1.23±3.42**		**26.51±8.54**		**0.64±2.28**	
	**≥30**	**28.15±6.15**		**1.32±3.38**		**26.35±8.36**		**0.48±2.00**	
**Alcohol consumption**	**No**	**28.40±6.01**	**0.48**	**1.29±3.40**	**0.89**	**26.56±8.34**	**0.95**	**0.54±2.09**	**0.06**
	**Yes**	**28.94±5.62**		**1.23±4.19**		**26.52±9.29**		**1.18±3.31**	

*independent t-test or one way ANOVA (Where applicable); ** Mann‒Whitney U test or Kruskal‒Wallis test (Where applicable).

In the regression model, the relationship between DMFT and the examined variables (age, gender, marital status, level of education, WSI, chronic diseases (such as diabetes, hypertension, stroke, cardiovascular diseases, cancer, chronic obstructive pulmonary diseases), smoking, alcohol consumption and body mass index) was evaluated, and no significant relationship was found (Table 3).

**Table 3:** The association between DMFT index and demographic factors, chronic diseases and behavioral habits in the elderly population of the Azar cohort.

Variable	Group	IRR*	95%CI	P
**Age**	**65–70**	**1.03**	**0.95–1.11**	**0.48**
	**60–64**	**Ref**		
**Gender**	**Female**	**1.03**	**0.92–1.15**	**0.57**
	**Male**	**Ref**		
**Marital status**	**Married**	**1.00**	**0.89–1.13**	**0.94**
	**unmarried**	**Ref**		
**Education level**	**Illiterate**	**1.23**	**0.98–1.54**	**0.07**
	**Primary**	**1.20**	**0.96–1.50**	**0.11**
	**Diploma**	**1.15**	**0.92–1.44**	**0.21**
	**University**	**Ref**		
**WSI** **(Wealth Score Index)**	**Very poor**	**1.02**	**0.88–1.18**	**0.81**
	**Poor**	**1.03**	**0.89–1.20**	**0.68**
	**Medium**	**1.02**	**0.88–1.18**	**0.81**
	**Good**	**1.01**	**0.85–1.20**	**0.91**
	**Very good**	**Ref**		
**Diabetes mellitus**	**Yes**	**0.98**	**0.89–1.08**	**0.80**
	**No**	**Ref**		
**Hypertension**	**Yes**	**0.99**	**0.91–1.08**	**0.91**
	**No**	**Ref**		
**Stroke**	**Yes**	**0.99**	**0.76–1.29**	**0.96**
	**No**	**Ref**		
**Cardiovascular diseases**	**Yes**	**0.99**	**0.83–1.13**	**0.98**
	**No**	**Ref**		
**Cancer**	**Yes**	**0.97**	**0.68–1.40**	**0.90**
	**No**	**Ref**		
**Depression**	**Yes**	**0.99**	**0.89–1.10**	**0.88**
	**No**	**Ref**		
**Chronic obstructive pulmonary diseases**	**Yes**	**1.00**	**0.85–1.18**	**0.91**
	**No**	**Ref**		
**Smoking**	**Smoker**	**1.10**	**0.95–1.26**	**0.18**
	**Ex-smoker**	**1.03**	**0.90–1.18**	**0.60**
	**Non-smoker**	**Ref**		
**BMI (kg/m2)**	**<18.5**	**1.06**	**0.71–1.60**	**0.78**
	**25–29.9**	**0.98**	**0.89–1.09**	**0.77**
	**30 ≤**	**0.97**	**0.87–1.08**	**0.61**
	**18.5–24.9**	**Ref**		
**Alcohol consumption**	**Yes**	**1.01**	**0.78–1.31**	**0.92**
	**No**	**Ref**		

*Incidence rate ratio

## Discussion

The present study evaluated dental caries and associated factors in the elderly population of the Azar cohort. The results indicated a high prevalence of edentulism and a high DMFT score among elderly individuals. Although the Kruskal-Wallis test showed significant mean differences in DMFT based on certain variables, no significant relationship was found between DMFT and the variables considered in the multiple regression analysis.

In the present study, the mean DMFT score for elderly individuals was 28.42. This finding is comparable to the study conducted by Meibodi et al. in Yazd city, which reported a mean DMFT score of 26.6 [7], as well as the research by Freitas et al. in Brazil in 2016 which found a score of 28.16 [8], and the study by Ziebolz et al. in Germany in 2017 which reported a mean DMFT of 26.4 for elderly individuals [9]. However, other studies have yielded different results. According to the national dental survey conducted in Iran in 2013, the mean DMFT score for elderly individuals was 25.71 [12]. Additionally, Tahani et al. in Isfahan [13] and Farokhnezhad Afshar et al. in Tehran [14] reported DMFT score of elderly individuals as 20.5 and 22, respectively. The discrepancies in these findings may be attributed to the varying settings of the studies. The participants in the Isfahan study were elderly individuals attending municipal centers, and those in Tehran were elderly individuals frequenting parks. Those who engage in outdoor activities or sports tend to be more active and healthier.

In the present study, 70.8% of the elderly participants were edentulous. According to a national dental survey conducted in Iran, 50% of elderly individuals were reported to be edentulous [12]. Freitas et al. (2016) reported that 50.8% of elderly individuals in Brazil were edentulous [8]. The higher prevalence of edentulism in the Azar cohort may be attributed to cultural differences in the Azerbaijan province.

In the current study, 69.30% of the elderly population had full dentures, which aligns with the findings of a national dental survey in Iran that reported a prevalence of 52% [12]. Additionally, only 2.5% of the elderly had an unmet need for full dentures. In Iran, certain insurance plans cover full denture treatments, which may impact these statistics.

In the present study, no significant relationship was observed in the regression analusis between DMFT index and increasing age among elderly individuals. This finding contrasts with the results of a study conducted in Yazd [7]. The discrepancy may be attributed to the age range of the participants in Yazd, which was 61–102 years old.

In the present study, consistent with the findings of Meibodi et al.(2016) in Yazd [7] and Angelis et al.(2015) in Italy [11], no significant relationship was observed between the DMFT index and gender. However, a study conducted by Andari et al. in Beirut in 2015 revealed a higher DMFT index among elderly women [20]. This discrepancy may be attributed to cultural differences and the inclusion of participants from social organizations and primary healthcare centers in Beirut and its suburbs.

In the current study, no association was found between DMFT index and educational level. This finding contrasts with the results reported by Andari et al. in Beirut, which indicated that highly educated elderly individuals had lower DMFT scores [20]. This discrepancy may be attributed to adjustment for several confounding variables in our analysis. As shown in Table 2, a simple analysis revealed that the mean DMFT and its components exhibited a significant differences based on educational level. Additionally, the study conducted by Wang et al. in 2017 in China found that elderly individuals with low socioeconomic status experienced poor oral health [21], which is inconsistent with the results of the present study. These associations can be partly explained partly by factors such as diet, behavior, and awareness particularly in rural areas.

### Strengths and limitations

As a strength point, the present study represents the first comprehensive assessment of DMFT index and its related factors in elderly individuals in northwestern Iran, utilizing a large sample size. Consequently, the results can be generalized to the population of northwestern Iran. However, it is important to note that a cross-sectional study design cannot establish causality, which is a limitation of this research. Additionally, the lack of data regarding periodontal status in the Azar cohort restricts the assessment of overall oral health status. Therefor, it is recommended that future studies considered both the DMFT index and the periodontal status. Another limitation is that, within the Azar cohort, only one individual had their DMFT index assessed through oral examination, and the cohort did not calculate the level of agreement for this assessment.

## Conclusion

The high prevalence of edentulism and the significant rate of missing teeth amongthe elderly population in the Azar cohort highlight the poor dental health status of older individuals. It is crucial to integrate oral health programs with general health initiatives. Public health policies aimed at improving oral health in adolescents and young adults should be implemented to help preserve a greater number of healthy, and natural teeth into old age. Enhancing access to dental services, including insurance coverage and the incorporation of dental care within the primary health care system, can be highly beneficial.

## Supporting information

S1 Checklist STROBE statement—checklist of items that should be included in reports of *cross-sectional studies*. (DOC)

## Abbreviations

DMFT: Decayed, Missing, and Filled Teeth in the permanent dentition
DT: number of Decayed Teeth in the permanent dentition)
MT: number of Missing Teeth due to caries in the permanent dentition
FT: number of Filled Teeth in the permanent dentition
PERSIAN: Prospective Epidemiological Research Studies in Iran
BMI: Body Mass Index
IRR: Incidence Rate Ratio
